# Virtual audits of the urban streetscape: comparing the inter-rater reliability of GigaPan® to Google Street View

**DOI:** 10.1186/s12942-020-00226-0

**Published:** 2020-08-12

**Authors:** Katherine N. Bromm, Ian-Marshall Lang, Erica E. Twardzik, Cathy L. Antonakos, Tamara Dubowitz, Natalie Colabianchi

**Affiliations:** 1 School of Kinesiology, University of Michigan, 1145 Observatory Lodge, 1402 Washington Heights, Ann Arbor, MI 48109-2013 USA; 2 Department of Epidemiology, School of Public Health, University of Michigan, 1415 Washington Heights, Ann Arbor, MI 48109 USA; 3 RAND® Corporation, 4570 Fifth Avenue, Suite 600, Pittsburgh, PA 15213 USA; 4 Institute for Social Research, University of Michigan, 426 Thompson St, Ann Arbor, MI 48104 USA

**Keywords:** GigaPan®, Google Street View, Reliability, Audit, Built environment

## Abstract

**Background:**

Although previous research has highlighted the association between the built environment and individual health, methodological challenges in assessing the built environment remain. In particular, many researchers have demonstrated the high inter-rater reliability of assessing large or objective built environment features and the low inter-rater reliability of assessing small or subjective built environment features using Google Street View. New methods for auditing the built environment must be evaluated to understand if there are alternative tools through which researchers can assess all types of built environment features with high agreement. This paper investigates measures of inter-rater reliability of GigaPan®, a tool that assists with capturing high-definition panoramic images, relative to Google Street View.

**Methods:**

Street segments (n = 614) in Pittsburgh, Pennsylvania in the United States were randomly selected to audit using GigaPan® and Google Street View. Each audit assessed features related to land use, traffic and safety, and public amenities. Inter-rater reliability statistics, including percent agreement, Cohen’s kappa, and the prevalence-adjusted bias-adjusted kappa (PABAK) were calculated for 106 street segments that were coded by two, different, human auditors.

**Results:**

Most large-scale, objective features (e.g. bus stop presence or stop sign presence) demonstrated at least substantial inter-rater reliability for both methods, but significant differences emerged across finely detailed features (e.g. trash) and features at segment endpoints (e.g. sidewalk continuity). After adjusting for the effects of bias and prevalence, the inter-rater reliability estimates were consistently higher for almost all built environment features across GigaPan® and Google Street View.

**Conclusion:**

GigaPan® is a reliable, alternative audit tool to Google Street View for studying the built environment. GigaPan® may be particularly well-suited for built environment projects with study settings in areas where Google Street View imagery is nonexistent or updated infrequently. The potential for enhanced, detailed imagery using GigaPan® will be most beneficial in studies in which current, time sensitive data are needed or microscale built environment features would be challenging to see in Google Street View. Furthermore, to better understand the effects of prevalence and bias in future reliability studies, researchers should consider using PABAK to supplement or expand upon Cohen’s kappa findings.

## Background

Research has shown a connection between the built environment (BE) where people live, work, and play and their physical, social, and mental health. A systematic review of the built environment and cardio-metabolic health found strong evidence of the association between the BE and a person’s physical health [1]. Leyden [2] found living in walkable, mixed-use neighborhoods was associated with greater social capital including a greater likelihood of trusting other people, getting to know neighbors, and involvement in one’s community. Urban neighborhood BE characteristics such as housing quality, exposure to greenspace, and other environmental conditions are also associated with psychological distress [3]. Although the body of evidence supporting the connection between the BE and health continues to grow, studying the BE continues to present unique methodological challenges.

In-person direct observation (DO) has been considered the gold standard when auditing features of the microscale BE [4]. The microscale environment is defined as built and social environment features representing neighborhood characteristics or details that are smaller in scale and are generally more likely to change over time with fewer costs [5]. This includes street-level environmental features like housing characteristics, sidewalk presence and conditions, street lighting, traffic control characteristics, intersection features, tree coverage, curb characteristics, graffiti, and trash. Similar to other researchers, we classify some microscale features as “finely detailed” [6]. This refers to features that are visually fine as a whole (e.g. presence of garbage, litter, or broken glass, presence of broken windows or bars on windows). Although DO is the gold standard in assessing the microscale environment, using DO can be costly and time intensive depending on the location and the size of the area being observed [7]. These limitations are especially problematic when the areas of interest are geographically dispersed across various political or administrative divisions (e.g. states, provinces, prefectures) or countries.

Google Street View (GSV) has been used as a reliable tool to observe the microscale BE and is a cheaper alternative to DO [6, 8]. GSV provides an individual with a panoramic, 360° view from a selected street, with the ability to move along the street on a computer and adjust zoom settings. While GSV has been used reliably to study BE features, it has also presented its own set of limitations. This includes limitations that are dependent upon the method used to assess features in the GSV imagery (e.g. human auditors vs. deep-learning technologies) and limitations that are relevant regardless of the method used. In one study that used human auditors to code features in GSV imagery, reliability was not as high when considering finely detailed features, such as the presence of litter, or when recording qualitative observations, such as the quality of sidewalk or housing [6]. Using human auditors to assess streetscape characteristics can also be subjective and costly for large-scale studies [9].

Deep-learning technologies have advanced our abilities to widely and objectively assess street features by making use of the pixels in GSV imagery. Studies have used machine learning to parse pixels of GSV imagery into different categories (e.g., sky, trees, and buildings) to generate very precise estimates of features [9, 10]. For example, Yin and Wang [9] generated the proportion of sky in GSV images and found it was negatively correlated with pedestrian activity and walkability. Researchers have also used GSV and a combination of other 2D and 3D data sources to make 3D GIS models and examine microscale urban design characteristics related to physical activity and pedestrian behavior [11]. Building 3D GIS models allows the user to interactively assess details of the streetscape from many angles, points, or locations [11]. Therefore, these models may improve researchers’ abilities to objectively assess features of the urban streetscape. 3D GIS models can be built using open source software and there have been many advances in procedural modeling, computer vision, and photogrammetry that make this process easier [11]. However, the application of these principles requires knowledge of artificial intelligence (e.g., machine learning, computer vision) and the computational skills and capacity to implement them.

Furthermore, although GSV is available for various cities throughout the world, GSV imagery is not available for every street in many countries, including many developed countries [12]. In the United States (USA), imagery is updated irregularly, with urban areas tending to have more complete coverage and more frequent updates than rural areas [6]. These limitations could prove problematic for studies in which the imagery date is crucial to the study’s aims, for studies across varying levels of urbanicity/rurality, or for studies in countries with sparse imagery availability. Another problem unique to GSV is the variation in imagery dates on a single street segment. As the auditor navigates the street in GSV, the latest imagery available may vary for different portions of the segment. It can be challenging to control for this variation; and therefore, the fluctuating dates have the potential to introduce error into an audit that is meant to represent the BE at a specific point in time [13].

Using GigaPan® to audit the BE is one potential solution to some of the shortcomings of using GSV or DO for BE audits. GigaPan® is a tool that assists in capturing high-definition panoramic images, and its use auditing the BE is underexplored. More specifically, GigaPan® is a robotic camera mount that can be used with most digital single-lens reflex cameras to capture panoramas composed of billions of pixels. After placing a camera on the GigaPan® apparatus, the GigaPan® apparatus is then attached to a tripod to allow for ease of use and to improve the vantage point. The GigaPan® mount maneuvers the camera to take hundreds to thousands of photographs of the designated area. Then, the images are uploaded and are stitched together using the GigaPan® Stitch Software that is downloaded onto a computer. This results in a detailed, enhanced panorama with increased zoom and improved resolution. Compared to GSV, GigaPan® allows for greater control of the temporal aspect of capturing and using images in real time as the researcher is responsible for deciding when and where images are taken. GigaPan® has been used for rangeland monitoring of natural resources in ecological studies, for analyzing the community structure of ants in Costa Rica, and by the National Aeronautics and Space Administration for planetary analog field experiments [14–16].

In recent research, our study team examined the reliability of GigaPan® as a method for assessing park characteristics, as well as the validity of using GigaPan® to measure park attributes in comparison to GSV and DO [17]. This research showed GigaPan® was a reliable method for collecting data on park attributes and a comparably valid method to GSV and DO [17]. Research by our study team also documented the validity of GigaPan® imagery in assessing street segment BE attributes [18]. Using DO as the gold standard, our findings for street segments showed GigaPan® audits obtained comparably valid results relative to GSV [18]. However, the reliability of using GigaPan® to document street segment BE characteristics has yet to be established. We hypothesized GigaPan® may be better suited for measuring microscale BE features compared to GSV. Given the potential benefits of using GigaPan® to capture the BE, this study seeks to ascertain and compare the inter-rater reliability (IRR) of BE constructs coded by multiple, human auditors using GigaPan® and GSV imagery.

## Methods

### Study sample

This study is an extension of the Pittsburgh Hill/Homewood Neighborhood Change and Health (PHRESH) study. The PHRESH study is led by RAND with the University of Michigan as a collaborator. It is an ongoing community-focused research study examining connections between features of the built and social environment and health in two, low-income neighborhoods (the Hill District and Homewood) in Pittsburgh, Pennsylvania, USA whose residents are predominately African American. Additional details regarding neighborhood selection can be found elsewhere [19]. This GigaPan® and GSV ancillary study was led by the University of Michigan to document street-scale features using GigaPan® that could be associated with walking, physical activity, and obesity. Briefly, RAND collected and electronically stitched the images to complete the GigaPan® panoramas, while the University of Michigan used human auditors to audit the GigaPan® panoramas, the GSV imagery, and analyze the data.

To assess the street environments in these two neighborhoods, a random sample of 614 unique street segments, approximately 25% of the street segments in each of the two neighborhoods, were audited. Of the 614 street segments, 20% (n = 124) were randomly selected, coded by two raters, and included in this study of IRR. Thus, the reliability sample represents both neighborhoods: Homewood and the Hill District. All selected street segments were audited using GSV imagery and GigaPan® imagery. Both types of imagery were coded by human auditors who reviewed the imagery and determined the presence, quality, or quantity of a BE feature along the streetscape. Details regarding image capture and coding are described below.

### Capturing street imagery using GigaPan®

RAND field staff recruited and trained neighborhood residents of Pittsburgh, Pennsylvania to capture images of the selected street segments using the GigaPan® apparatus. Before entering the field, staff completed training that required them to read the GigaPan® manual, watch a demonstrational video on the use of the GigaPan® apparatus, and take practice photos to become comfortable using the GigaPan® equipment.

**Fig. 1:**
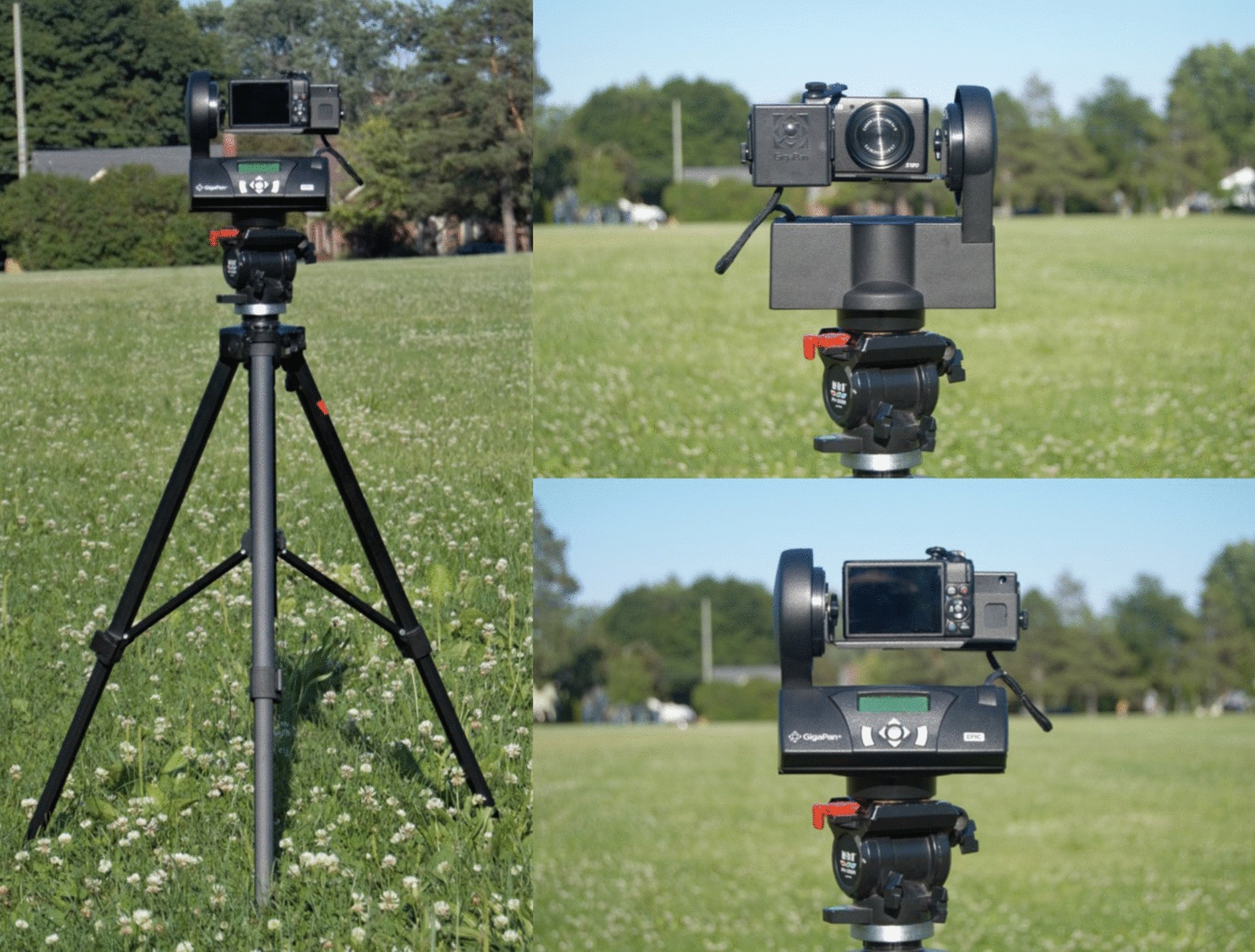
Canon PowerShot® S120 camera mounted on the GigaPan® Epic and secured on a tripod

The GigaPan® apparatus is a camera mount consisting of a panoramic tripod head that holds and stabilizes a digital camera of the user’s choice. In this study, we mounted a Canon® PowerShot S120 camera onto the GigaPan® Epic apparatus and secured it on a tripod (see Fig. 1). With the camera secured on the tripod, the user aimed the device to the upper-left corner and lower-right corner to set the boundaries of the area they wanted to capture. The built-in software of the GigaPan® apparatus then computed the number of images needed to complete the panorama. Next, the user pressed the shutter-release button and the apparatus captured the individual images which were later assembled into a larger panoramic image using the GigaPan® Stitch Software on a computer.

**Fig. 2:**
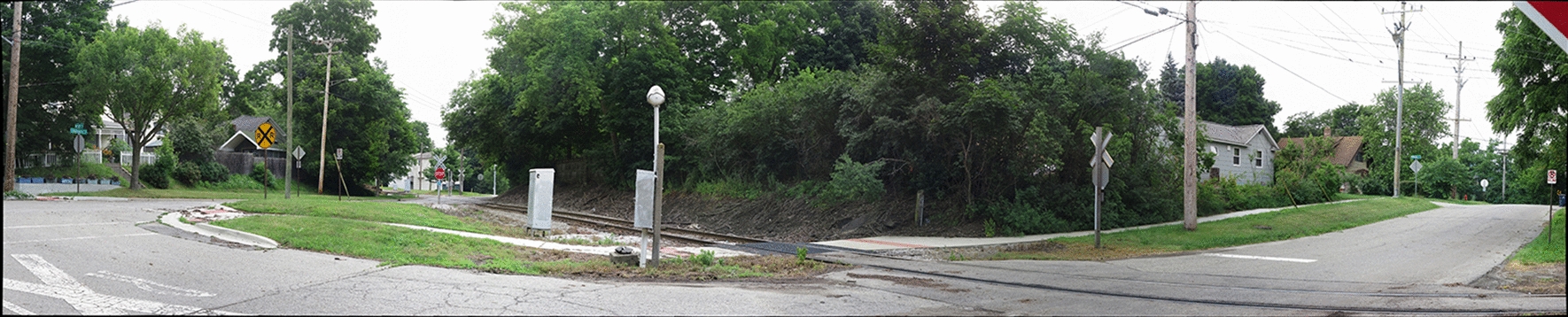
A GigaPan® panorama for a non-study street segment in Ann Arbor, Michigan, USA. The panorama is the result of many smaller images that were stitched together electronically using the GigaPan® Stitch Software. The individual images composing the panorama were captured by a Canon® PowerShot S120 mounted on a GigaPan® Epic that was secured on a tripod. The number of pixels in this image was reduced dramatically to meet journal file size requirements. Additional interactive photos shared by other users of GigaPan® can be accessed on the GigaPan® website via URL [20]

RAND field staff took a GigaPan® panorama for both sides of the selected street segments. After the images were taken, RAND used the GigaPan® Stitch Software to electronically stitch the individual images into a high-resolution panorama. A resulting panoramic image for a non-study location can be seen in Fig. 2. The panoramic GigaPan® photos were then sent to the University of Michigan so that trained, human auditors could code various BE features identified within each panoramic photo.

### Coding the GSV and GigaPan® imagery

University of Michigan auditors performed GSV audits using Google Earth Pro version 7.3. All study street segments were saved in the form of KMZ files for use in Google Earth. Each street segment had a unique segment ID that served as the identifier. Example GSV imagery corresponding to the non-study street segment shown in Fig. 2 can be accessed via URL [21]. To document street-scale features that could be associated with walking, physical activity, and obesity, the University of Michigan auditors coded the GSV imagery and GigaPan® imagery separately using a modified, electronic version of the Bridging the Gap Community Measures Project (BTG-COMP) auditing tool [22]. This tool is formally known as the BTG-COMP Street Segment Observation Form and was designed to assess neighborhood street-level features related to physical activity. Based on previous pilot study research that used in-person DO as the auditing method, the tool had good reliability [23]. Additional details regarding the selection of the measures included in the tool can be found elsewhere [23]. In brief, a socio-ecological framework guided the overall development of the original BTG-COMP auditing tool, and empirical literature and an expert panel were used to identify features of the neighborhood environment associated with physical activity. The tool focused on features that could not be assessed using available national data. Moreover, our study team made modifications to the original tool to fit the local context of the two neighborhoods we audited. For example, we added a question about the slope of the segment because the Hill District and Homewood neighborhoods are hilly and the steepness of the street segments was expected to be related to neighborhood residents’ physical activity behaviors. We also developed a modified coding manual to include the new items which was used as the basis for training auditors before they began auditing street segments using GigaPan® and GSV imagery.

The training process for coding both types of imagery consisted of reviewing the coding manual as a group, reading the manual individually, experimenting with GSV and GigaPan® technology, and then completing multiple practice audits with both GigaPan® imagery and GSV imagery. Practice audits used locations in various urban cities of the USA that were outside of the study area. The locations were selected to help the auditors become familiar with coding features and amenities expected to be seen in Pittsburgh, Pennsylvania. During training, weekly meetings were held to discuss coding discrepancies within the group. Eighty percent agreement or higher was needed to be certified as an auditor, which was calculated between each auditor’s coded answers and the lab manager.

After training was complete, coding of the 614 unique street segments for both GSV and GigaPan® began. Two independent, trained auditors double-coded a random sample of the 614 street segments. One hundred twenty-four street segments served as the reliability sample for this study. The same street segment was coded by two different auditors for both GSV and GigaPan® audits. All GSV imagery was the most current imagery available at the time audits were completed. GSV imagery dates ranged from July 2007 to September 2016, while GigaPan® photos were taken from September 2015 to December 2015. The majority of GSV audits were completed using imagery from 2016.

### Quality control

Prior to analysis, we evaluated the accuracy and quality of GigaPan® and GSV imagery for each of the randomly-selected reliability segments. Eighteen segments were dropped from the analysis sample. Among the 18 segments, 13 were dropped for having GigaPan® imagery or collection issues, one segment was dropped for having a GSV imagery or collection issue, and four segments were dropped for having both GigaPan® and GSV imagery and/or collection issues. Dropped segments had at least one issue. The GSV imagery issues included the following: poor image quality, the incorrect cross street was used, the segment analyzed was not a street, the imagery was incomplete, or the street view imagery did not exist for the segment. The GigaPan® imagery issues included the following: the imagery file could not be opened, the image only covered a small fraction of the segment, or the image was taken in the wrong location. The resulting reliability analysis sample included 106 segments with complete imagery for GigaPan® and GSV.

In order to estimate the prevalence, features with three response options were recoded into two categories, with “1” indicating presence of the feature on one or both sides of the street and “0” indicating absence of the feature. The prevalence of each feature was then estimated. Features with a prevalence below 0.10 in either the GigaPan® or GSV sample were excluded from the analysis given the poor performance of Cohen’s kappa and the prevalence-adjusted bias-adjusted kappa (PABAK) when prevalence of a feature is extremely low [24–26]. After eliminating those features with a prevalence below 0.10, 28 features were retained for the analysis. We collapsed the 28 features into three concepts: land use, traffic and safety, and public amenities. Table 1 outlines each concept, the features comprising each concept, the question used to assess the feature, and the original response options. In general, land use includes features of residential buildings, nonresidential buildings, tree coverage, and segment slope. Traffic and safety includes sidewalk presence, sidewalk continuity, sidewalk buffers, traffic lighting, street and sidewalk lighting, crosswalk presence, curbs, and lane attributes. Public amenities includes transit features, trash maintenance, aesthetics, sidewalk condition, and perceived safety.

**Table 1:** The 28 features retained for reliability analysis after dropping features with a prevalence below 0.10

Feature	Question asked on coding form	Response options on coding form
*Land use concept*		
Detached housing	Scan both sides of the street for the presence of: detached housing	No, Yes on One Side, or Yes on Both Sides [of the street]
Institutional buildings	Scan both sides of the street for the presence of: institutional buildings	No, Yes on One Side, Yes on Both Sides [of the street]
Broken or boarded windows	Are there any broken/boarded windows?	No, Yes
Attached housing	Scan both sides of the street for the presence of: attached housing	No, Yes on One Side, Yes on Both Sides [of the street]
Trees that shade sidewalk	Do trees shade sidewalk?	None/Few, Some, Many
Amount of street trees	How many street trees are there?	None/Few, Some, Many
Bars on the windows	Are there any bars on windows?	No, Yes
Slope of the segment	What is the slope of the segment?	Flat, Slight Hill, Steep Hill
Vacant building or lot	Scan both sides of the street for the presence of: vacant building or lot	No, Yes on One Side, Yes on Both Sides [of the street]
Housing apartments	Scan both sides of the street for the presence of: housing apartments	No, Yes on One Side, Yes on Both Sides [of the street]
*Traffic and safety concept*		
Stop sign	Is there a stop sign?	No, Yes
Sidewalk	Is there any sidewalk?	No, Yes on One Side, Yes on Both Sides [of the street]
Marked crosswalk	Is there a marked crosswalk?	No, Yes
Traffic light	Is there a traffic light?	No, Yes
Curb	Is there any curb?	No, Yes on One Side, Yes on Both Sides [of the street]
Number of traffic lanes	Number of lanes of vehicular traffic	[Open Response]
Street or sidewalk lighting	Is there any street or sidewalk lighting?	No, Yes on One Side, Yes on Both Sides [of the street]
Continuous sidewalk	Is there any continuous sidewalk?	No, Yes on One Side, Yes on Both Sides [of the street]
Street and sidewalk buffer	Is there a street and sidewalk buffer?	No, Yes on One Side, Yes on Both Sides [of the street]
Continuous sidewalk on both ends	Is there any continuous sidewalk at both ends between segments?	No, Yes on One Side, Yes on Both Sides [of the street]
Missing curb cuts at crossing	Are there any curb cuts or ramps missing at crossing points?	No, Yes on One Side, Yes on Both Sides [of the street]
*Public amenities concept*		
Bus stop	Is there a bus stop?	No, Yes
Public trash can	Is there a public trash can?	No, Yes
Perceived safety of segment	How safe do you feel walking on this segment?	Unsafe/Not Very Safe, Pretty Safe/Very Safe
Overall condition of sidewalk	What is the overall condition of the sidewalk?	Poor, Moderate, Good, Under Repair
Garden, flower bed, or planter	Is there a garden, flower bed, or planter?	No, Yes
Amount of trash on street	What is the amount of trash/litter on the street?	None, A Little, Some, A Lot
Attractiveness for walking	Overall, how attractive would you rate this segment for walking?	Unattractive, Neutral, Attractive

Ten features were retained in the land use concept, 11 features in the traffic and safety concept, and seven features in the public amenities concept

### Statistical analysis

Average prevalence (PR) was calculated for each feature. In this study, this statistic represents the proportion of street segments containing the feature of interest. Percent agreement (PA), Cohen’s kappa, and PABAK were also calculated for each feature with valid GigaPan® and GSV audit data. Although prevalence was estimated using the dichotomized versions of each of the features, the reliability statistics (PA, Cohen’s kappa, and PABAK) were estimated using the original response options. The PA represents the total number of segments for which the two auditors selected the same response option (e.g., both auditors agreed the feature was present or both auditors agreed the feature was absent for the segment) divided by the total number of segments audited. This is then converted to a percentage to get the PA. The PA is a direct measure rather than an estimate; and therefore, confidence intervals (CI) are not needed [27]. The shortcoming of this statistic is that it does not account for the possibility that some agreement would be due to chance [27]. Thus, Cohen’s kappa is a measure of agreement that corrects for agreement due to chance [28]. Although Cohen’s kappa is the primary IRR statistic used in research, it can be affected by bias between auditors and the level of prevalence of the feature being observed [24]. Therefore, PABAK was also calculated. PABAK is a kappa statistic that accounts for prevalence and bias by holding prevalence constant at 0.50. Cohen’s kappa and PABAK values are most similar when prevalence is close to 0.50. Additionally, PABAK is not recommended in instances of extremely low prevalence. However, by eliminating variables where the prevalence was below 0.10, we were able to overcome this limitation [26]. Cohen’s kappa and PABAK are both estimated statistics with ranges from − 1 to + 1. A value of + 1 represents perfect agreement, a value of 0 represents agreement due to chance, and a value of − 1 represents perfect disagreement. The scale used for assessing the level of IRR based on Cohen's kappa and PABAK values is widely used in reliability literature and is as follows: poor (< 0.00), slight (0.00–0.20), fair (0.21–0.40), moderate (0.41–0.60), substantial (0.61–0.80), and almost perfect (0.81–1.0) [29]. As noted by Landis and Koch, the divisions/ranges are arbitrary but provide useful benchmarks for discussion, and the nomenclature also provides consistency for describing the relative strength of agreement [29]. For each BE feature, CIs were also calculated for Cohen’s kappa and PABAK given both statistics are estimates of the reliability based on the sample and the CIs provide a range of likely values for the estimate. The CIs were evaluated to determine differences between the two audit methods. Non-overlapping confidence intervals represented a statistically significant difference. All analyses were conducted using Stata version 14.2.

## Results

Across the three BE concepts (land use, traffic and safety, and public amenities), some features had missing data and some features were only relevant if another feature was present. Five features were only relevant if another feature was present and thus followed skip logic to ensure they were not audited. For example, the audit tool question ascertaining the continuity of the sidewalk was only answered if the auditor indicated a sidewalk was present. If a sidewalk was not marked as present, the continuous sidewalk question was not answered. After taking into account missing data and skip logic, sample sizes ranged from 79 to 106.

Overall when using GSV, eleven of the 28 BE features had Cohen’s kappa values in the substantial to almost perfect reliability range (*the presence of: detached housing, institutional land use, a stop sign, a sidewalk, a marked crosswalk, a traffic light, a continuous sidewalk, a continuous sidewalk (both ends), a bus stop, and a public trash can; the number of traffic lanes*), 12 features had kappa values in the fair to moderate reliability range (*the presence of: broken/boarded windows, attached housing, trees that shade the sidewalk, bars on the windows, a vacant building/lot, housing apartments, a curb, street or sidewalk lighting, a street and sidewalk buffer, and a garden, flower bed, or planter; the amount of street trees; the slope of the segment*), and five features had kappa values within the poor to slight reliability range (*the absence of curb cuts at a crossing; the perceived safety of the segment; the overall condition of the sidewalk; the amount of trash on the street, and attractiveness for walking*). On the other hand, GigaPan® had seven BE features within the substantial to almost perfect reliability range (*the presence of: a stop sign, a sidewalk, a marked crosswalk, a traffic light, a curb, and a bus stop; the number of traffic lanes*), 16 features within the fair to moderate reliability range (*the presence of: detached housing, institutional land use, broken/boarded windows, attached housing, trees that shade the sidewalk, bars on the windows, street or sidewalk lighting, a continuous sidewalk, a street and sidewalk buffer, a continuous sidewalk (both ends), a public trash can, and a garden, flower bed, or planter; the amount of street trees; the perceived safety of the segment; the overall condition of the sidewalk; the amount of trash on the street*), and five features within the poor to slight reliability range (*the presence of: a vacant building/lot, housing apartments; the slope of the segment, the absence of curb cuts at a crossing; the attractiveness for walking*).

After adjusting for the effects of bias and prevalence, GSV and GigaPan® had more features in the upper reliability ranges. GSV had 16 BE features with PABAK values in the substantial to almost perfect reliability range (*the presence of: detached housing, institutional land use, attached housing, bars on the windows, housing apartments, a stop sign, a sidewalk, a marked crosswalk, a traffic light, a continuous sidewalk, a continuous sidewalk (both ends), a bus stop, and a public trash can; the slope of the segment; the number of traffic lanes; the absence of curb cuts at a crossing*), eight features in the fair to moderate reliability range (*the presence of: trees that shade the sidewalk, a vacant building/lot, a curb, street or sidewalk lighting, a street and sidewalk buffer, and a garden, flower bed, or planter; the amount of street trees; the absence of curb cuts at a crossing*), and four features in the poor to slight reliability range (*the perceived safety of the segment; the overall condition of the sidewalk; the amount of trash on the street; the attractiveness for walking*). Using GSV, 17 features had PABAK values in the substantial to almost perfect reliability range (*the presence of: institutional land use, broken/boarded windows, attached housing, trees that shade the sidewalk, housing apartments, a stop sign, a sidewalk, a marked crosswalk, a traffic light, a curb, street or sidewalk lighting, a continuous sidewalk, a street and sidewalk buffer; slope of the segment, a bus stop, and a public trash can; the number of traffic lanes*) and 11 features had PABAK values in the fair to moderate reliability range (*the presence of: detached housing, bars on the windows, a vacant building/lot, a continuous sidewalk (both ends), and the presence of a garden, flower bed, or planter; amount of street trees; the absence of curb cuts at a crossing; the perceived safety of the segment; the overall condition of the sidewalk; the amount of trash on the street; the attractiveness for walking*). None of the features assessed with GigaPan® had PABAK values in the poor to slight reliability range. The PA ranged from 27% (attractiveness for walking) to 98% (presence of a street light) using GSV, while the PA ranged from 47% (attractiveness for walking) to 95% (presence of a bus stop) using GigaPan®. The PA values were consistently higher than Cohen’s kappa and PABAK values. Reliabilities across the three broad concepts (i.e., land use, traffic and safety, and public amenities) were varied, as described below.

### GigaPan®

None of the ten land use features (*presence of detached housing, presence of institutional land use, presence of broken/boarded windows, presence of attached housing, presence of trees that shade the sidewalk, the amount of street trees, presence of bards on the windows, the slope of the segment, the presence of a vacant building/lot, presence of housing apartments*) evaluated using GigaPan® reached the substantial to almost perfect agreement range based on Cohen’s kappa (Table 2). However, presence of broken/boarded windows, presence of attached housing, presence of housing apartments, presence of trees that shade the sidewalk, and the slope of the segment had PABAK reliability values in the substantial range and presence of institutional land use had a PABAK value in the almost perfect range.

**Table 2:** Land use features for street segments

Features	N	PR		PA		Cohen’s kappa				PABAK			
		GP	GSV	GP (%)	GSV (%)	GP	95% CI	GSV	95% CI	GP	95% CI	GSV	95% CI
Detached housing	106	0.50	0.59	74	76	0.58	[0.44, 0.71]	0.64	[0.52, 0.77]	0.60	[0.48, 0.73]	0.65	[0.52, 0.77]
Institutional	106	0.13	0.18	90	91	0.54	[0.30, 0.79]	0.69	[0.52, 0.87]	0.84	[0.76, 0.93]	0.86	[0.77, 0.94]
Broken/boarded windows*	105	0.22	0.35	81	80	0.45	[0.25, 0.65]	0.56	[0.39, 0.73]	0.62	[0.47, 0.77]	0.60	[0.44, 0.76]
Attached housing	105	0.20	0.30	80	77	0.41	[0.21, 0.60]	0.50	[0.38, 0.58]	0.70	[0.58, 0.82]	0.66	[0.53, 0.78]
Trees that shade sidewalk	105	0.17	0.32	82	67	0.37	[0.17, 0.57]	0.31	[0.17, 0.46]	0.73	[0.62, 0.84]	0.50	[0.36, 0.64]
Amount of street trees	106	0.38	0.45	63	59	0.33	[0.18, 0.48]	0.33	[0.20, 0.47]	0.45	[0.31, 0.59]	0.39	[0.25, 0.53]
Bars on the windows*	105	0.19	0.20	76	82	0.26	[0.08, 0.45]	0.43	[0.22, 0.64]	0.52	[0.36, 0.69]	0.64	[0.49, 0.79]
Slope of the segment	106	0.17	0.14	75	83	0.19	[0.01, 0.38]	0.30	[0.09, 0.52]	0.63	[0.51, 0.76]	0.75	[0.64, 0.85]
Vacant building/Lot	106	0.30	0.45	58	57	0.13	[0.06, 0.27]	0.25	[0.10, 0.40]	0.38	[0.23, 0.52]	0.35	[0.21, 0.49]
Housing apartments	106	0.16	0.20	75	75	0.15	[−0.02, 0.33]	0.24	[0.06, 0.41]	0.63	[0.51, 0.76]	0.62	[0.49, 0.74]

*PR* average prevalence, *PA *percent agreement, *GP *GigaPan®, *GSV *Google Street View, *CI *confidence interval, *PABAK *prevalence-adjusted bias-adjusted kappa. An * denotes the variable was dichotomous. All other variables not denoted with * were recoded to be dichotomous solely when calculating prevalence. Significant differences across audit tools are italicized.

Six of the eleven traffic and safety features (*presence of a stop sign, presence of a sidewalk, presence of a marked crosswalk, presence of a traffic light, presence of a curb, the number of traffic lanes*) had substantial to almost perfect agreement according to Cohen’s kappa (Table 3). Presence of a traffic light, presence of a curb, and the number of traffic lanes had Cohen’s kappa reliability values in the substantial range. Presence of a stop sign, presence of a sidewalk, and presence of a marked crosswalk fell into the almost perfect reliability range. Using PABAK, presence of a curb and the number of traffic lanes remained in the substantial range, while the presence of a traffic light was measured with almost perfect agreement. Moreover, the presence of street or sidewalk lighting, the presence of a continuous sidewalk, and the presence of a street and sidewalk buffer could be measured with substantial reliability according to PABAK. Presence of a stop sign, presence of a sidewalk, and presence of a marked crosswalk had almost perfect agreement for both Cohen’s kappa and PABAK.

**Table 3:** Traffic and safety features for street segments

Feature	N	PR		PA		Cohen’s kappa				PABAK			
		GP	GSV	GP (%)	GSV (%)	GP	95% CI	GSV	95% CI	GP	95% CI	GSV	95% CI
Stop sign*	103	0.33	0.34	93	95	0.85	[0.74, 0.96]	0.89	[0.80, 0.99]	0.86	[0.76,0.97]	0.90	[0.82, 0.99]
Sidewalk	106	0.81	0.80	92	89	0.83	[0.73, 0.94]	0.76	[0.64, 0.89]	0.87	[0.79, 0.95]	0.83	[0.74. 0.92]
Marked crosswalk*	104	0.22	0.27	94	91	0.83	[0.70, 0.97]	0.78	[0.65, 0.92]	0.88	[0.79, 0.98]	0.83	[0.72, 0.94]
Traffic light*	104	0.16	0.14	93	98	0.75	[0.56, 0.93]	0.92	[0.81, 1.00]	0.87	[0.76, 0.97]	0.96	[0.91, 1.00]
Curb	106	0.78	0.73	86	70	0.68	[0.54, 0.82]	0.45	[0.31, 0.59]	*0.79*	*[0.69, 0.89]*	*0.55*	*[0.41, 0.68]*
Number of traffic lanes*	105	0.73	0.73	87	90	0.66	[0.50, 0.82]	0.76	[0.61, 0.90]	0.73	[0.60, 0.87]	0.81	[0.70, 0.92]
Street or sidewalk lighting	105	0.87	0.81	79	71	0.56	[0.40, 0.72]	0.44	[0.27, 0.61]	0.69	[0.57, 0.80]	0.57	[0.44, 0.70]
Continuous sidewalk	81	0.88	0.83	74	86	*0.43*	*[0.32, 0.50]*	*0.72*	*[0.57, 0.86]*	0.61	[0.46, 0.76]	0.78	[0.66, 0.90]
Street and sidewalk buffer	80	0.27	0.34	75	68	0.42	[0.21, 0.63]	0.35	[0.16, 0.54]	0.63	[0.48, 0.77]	0.51	[0.35, 0.67]
Continuous sidewalk (both ends)	81	0.75	0.78	65	80	*0.38*	*[0.32, 0.41]*	*0.63*	*[0.53, 0.78]*	0.48	[0.32, 0.64]	0.70	[0.57, 0.84]
Missing curb cuts at crossing	79	0.25	0.13	53	77	−0.17	[−0.20, −0.11]	0.06	[−0.04, 0.29]	*0.30*	*[0.13, 0.47]*	*0.66*	*[0.51, 0.80]*

*PR *average prevalence, *PA *percent agreement, *GP *GigaPan®, *GSV *Google Street View, *CI *confidence interval, *PABAK *prevalence-adjusted bias-adjusted kappa. An * denotes the variable was dichotomous. All other variables not denoted with * were recoded to be dichotomous solely when calculating prevalence. Significant differences across audit tools are italicized.

The assessments of the seven public amenities (*presence of a bus stop, presence of a public trash can, the perceived safety of the segment, the overall condition of the sidewalk, the presence of a garden, flower bed, or planter, the amount of trash on the street, and the attractiveness for walking*) were similar for both reliability statistics (Table 4). The presence of a bus stop was the only public amenity measured with substantial reliability. This feature was measured with almost perfect reliability after adjusting for the effects of bias and prevalence. In addition, using PABAK, the presence of a public trash can was measured with substantial reliability. All other public amenities were measured with slight, fair, or moderate reliability using Cohen’s kappa and PABAK.

**Table 4:** Public amenities features for street segments

Feature	N	PR		PA		Cohen’s kappa				PABAK			
		GP	GSV	GP (%)	GSV (%)	GP	95% CI	GSV	95% CI	GP	95% CI	GSV	95% CI
Bus stop*	104	0.13	0.10	95	97	0.79	[0.61, 0.97]	0.84	[0.66, 1.00]	0.90	[0.82, 0.99]	0.94	[0.87, 1.00]
Public trash can	103	0.14	0.11	83	93	0.33	[0.0, 0.57]	0.66	[0.42, 0.89]	0.67	[0.52, 0.82]	0.86	[0.76, 0.97]
Perceived safety of segment	106	0.57	0.75	65	56	*0.34*	*[0.20, 0.49]*	*-0.05*	*[*−*0.17, 0.08]*	0.30	[0.12, 0.49]	0.11	[−0.08, 0.31]
Overall condition of sidewalk	81	0.72	0.74	51	37	*0.29*	*[0.23, 0.46]*	*0.08*	*[*−*0.05, 0.20]*	0.25	[0.09, 0.43]	0.16	[0.02, 0.30]
Garden, flower bed, or planter*	105	0.22	0.25	77	77	0.34	[0.13, 0.55]	0.39	[0.19, 0.59]	0.54	[0.38, 0.71]	0.54	[0.38, 0.71]
Amount of trash on street	106	0.81	0.83	55	35	0.27	[0.13, 0.37]	0.10	[−0.02, 0.22]	*0.40*	*[0.27, 0.52]*	*0.13*	*[0.01. 0.26]*
Attractiveness for walking	106	0.43	0.57	47	27	0.14	[0.00, 0.30]	0.04	[−0.06, 0.14]	0.21	[0.06, 0.35]	−0.09	[−0.22, 0.04]

*PR *average prevalence, *PA *percent agreement, *GP* GigaPan®, *GSV *Google Street View, *CI* confidence interval, *PABAK *prevalence-adjusted bias-adjusted kappa. An * denotes the variable was dichotomous. All other variables not denoted with * were recoded to be dichotomous solely when calculating prevalence. Significant differences across audit tools are italicized.

### GSV

Presence of detached housing and the presence of institutional land use were measured with substantial agreement using GSV (Table 2). Presence of housing remained in the substantial range, while the presence of institutional land use was measured with almost perfect reliability, using PABAK. Presence of attached housing, presence of bars on windows, street slope, and presence of apartments also had substantial agreement based on PABAK.

Across all three BE concepts, the traffic and safety concept had the greatest number features in the substantial to almost perfect agreement range (Table 3). Presence of a sidewalk, presence of a marked crosswalk, number of traffic lanes, presence of a continuous sidewalk, and presence of a continuous sidewalk (both ends) had Cohen’s kappa values in the substantial range while presence of a stop sign and presence of a traffic light fell into the almost perfect range. The presence of a sidewalk, the presence of a marked crosswalk, and the number of traffic lanes were measured with almost perfect agreement using PABAK. The presence of missing curb cuts at a crossing was measured with substantial agreement while all other features measured with substantial to almost perfect agreement using PABAK mirrored those features falling within these respective ranges before adjustment (i.e., with Cohen’s kappa).

Public amenities had the lowest reliability for both Cohen’s kappa and PABAK (Table 4). Of the seven features assessed, presence of a public trash can was measured with substantial reliability and presence of a bus stop was measured with almost perfect reliability using Cohen’s kappa. Both of these features were measured with almost perfect agreement using PABAK. All other public amenities had reliability values in the poor, slight, fair, or moderate reliability ranges.

### GigaPan® vs. GSV

Comparing the confidence intervals presented in Tables 2–4, GigaPan® and GSV performed similarly (21 features had overlapping confidence intervals across the audit methods) with significant exceptions in the traffic and safety concept (Table 3) and public amenities concept (Table 4). Whether or not the sidewalk was continuous had higher agreement using GSV compared to GigaPan® (GSV: κ = 0.72, 95% Confidence Interval (CI): 0.57, 0.86 vs. GigaPan®: κ = 0.43, 95% CI: 0.32, 0.50), as did whether or not the sidewalk was continuous on both ends (GSV: κ = 0.63, 95% CI: 0.53, 0.78 vs. GigaPan®: κ = 0.38, 95% CI: 0.32, 0.41). GigaPan® was significantly more reliable (PABAK = 0.79, 95% CI: 0.69, 0.89) than GSV (PABAK = 0.55, 95% CI: 0.41, 0.68) in assessing the presence of a curb using PABAK. However, in assessing the presence of a curb cut at a crossing, GigaPan® was significantly less reliable (PABAK = 0.30, 95% CI: 0.13, 0.47) than GSV (PABAK = 0.66, 95% CI: 0.52, 0.80).

Within the public amenities concept, the perceived safety of the segment was more reliably assessed using GigaPan® (κ = 0.34, 95% CI: 0.20, 0.49) compared to Google Earth (κ = −0.05, 95% CI: −0.17, 0.08), as well as the overall condition of the sidewalk (GigaPan®: κ = 0.29, 95% CI: 0.23, 0.46 vs. GSV κ = 0.08, 95% CI: −0.05, 0.20). Additionally, in assessing the amount of trash on the street, GigaPan® was significantly more reliable (PABAK = 0.40, 95% CI: 0.27, 0.52) than GSV (PABAK = 0.13, 95% CI: 0.01, 0.26). Notably, all of these features within the public amenities concept were considered at best fairly reliable.

## Discussion

### *GigaPan*® *and GSV*

BE features in the traffic and safety concept were most reliably coded, based on Cohen’s kappa and PABAK, for both GSV and GigaPan®. Features in the traffic and safety concept were assessed for presence only, not quality. The objective nature of these questions made them easier for auditors to code and may be the reason reliability is highest for these features. Furthermore, across the auditing tools there were four significant differences in the level of agreement for BE features related to curbs and sidewalk continuity in the traffic and safety concept. Agreement was higher for identifying the presence of a curb using GigaPan® compared to GSV. However, there was lower agreement in identifying the presence of a missing curb cut at a crossing, continuous sidewalk, and continuous sidewalk (on both ends) using GigaPan® compared to GSV. This difference potentially stems from where the GigaPan® user positioned the camera on the street. Following the protocol, the user placed the camera on a tripod at the middle of the street segment. Although the camera rotates as images are captured, the camera does not traverse down the street as the GSV car does to capture imagery. Therefore, with GigaPan®, magnification of the imagery at the edge of the frame is reduced and slightly distorted because the object is farther away from the camera lens. This slight distortion would make it difficult for an auditor to reliably audit features at the end of the sidewalk/street (e.g. curb cuts at a crossing) and the continuity of the sidewalk as the auditor codes features towards the edges of the GigaPan® imagery frame (i.e. the ends of the sidewalk/street). In the future, a different lens could be used to reduce distortion and to increase the potential for these features to be coded reliably using GigaPan® imagery and/or multiple photos could be taken for longer street segments.

In the public amenities concept, GigaPan® performed significantly better than GSV across three features: the perceived safety of the segment, the overall condition of the sidewalk, and the amount of trash on the street. These are finely detailed BE features and are also more subjective. It is likely GigaPan® performed more reliably across this concept because of the enhanced detail capabilities the tool offers. However, despite the significant differences between GigaPan® and GSV across this concept, researchers should exercise caution in using GigaPan® to audit public amenities with reliability values below the moderate to almost perfect range. Researchers may consider utilizing a camera with different zoom capabilities and lens options on the GigaPan® apparatus to audit these BE features more reliably.

With regards to the land use concept, GigaPan® and GSV performed similarly. Agreement was low across many features (*presence of broken/boarded windows, presence of attached housing, presence of trees that shade the sidewalk, the amount of trees on the street, presence of bars on the windows, the slope of the segment, presence of a vacant building/lot, and the presence of housing apartments*); however, after accounting for the effects of bias and prevalence, many land use features demonstrated substantial or almost perfect IRR regardless of whether GigaPan® or GSV was used (*the presence of institutional land use, the presence of attached housing, the slope of the segment, and the presence of housing apartments*). The land use concept is a mixture of large-scale and finely detailed BE features. It includes large-scale features like the presence of trees and different types of housing, but also finely detailed features (e.g. the presence of broken windows and bars on windows) [6].

GSV performed similarly to previous studies using GSV audits to study the BE. In previous studies, GSV’s greatest limitations were assessing finely detailed features and features that involved making qualitative judgments [6, 7]. These limitations were also evident in our study across features like sidewalk quality and the presence of litter.

This is the first study to examine the IRR of assessing street segment BE features using GigaPan®. The findings are consistent with the GigaPan® IRR results found in our study of park BE features [17]. Overall, both studies found GigaPan® to be a reliable method to assess the BE. The results can be explained in part by the high-definition, panoramic GigaPan® images that are static and that our auditors perceived as easier to code. The enhanced detail in the GigaPan® images may also provide researchers with the ability to more reliably code finely detailed BE features.

Improvements in the technology of videos and photo stitching mobile phone apps offer additional ways to study the BE, but even with these new forms of technology, GigaPan® still has advantages. Videos are dynamic and provide new frames every millisecond, yet the time needed to code videos can be lengthy. Photo stitching apps for mobile phones provide users with the ability to take multiple photos and stich them together without special equipment; however, these apps do not consistently produce high quality panoramic images and may not meet the image quality standards required by researchers studying microscale features of the BE. As technology improves, these technologies may offer viable methods for assessing the BE.

Overall, GigaPan® and GSV performed similarly across the three BE concepts including features related to land use, traffic and safety, and public amenities. However, the three significant differences in the public amenities concept (the perceived safety of the segment, the overall condition of the sidewalk, and the amount of trash on the street) suggest GigaPan® may be better suited to assess finely detailed BE features. Yet, even with the high-resolution imagery produced by GigaPan®, these features only demonstrated fair reliability. Although the presence of litter reached moderate agreement in the GigaPan® IRR study of park BE features, the findings between the two studies are overall consistent as finely detailed features (e.g. presence of overgrowth and condition of open green space) had slight-to-fair reliability in the park study [17]. Therefore, such features may require high-resolution imagery not currently available through GSV, but could be produced by using a camera with different zoom capabilities and lens options on the GigaPan® apparatus. Additionally, although there was higher IRR using GSV imagery compared to GigaPan® imagery for three features in the traffic and safety concept (i.e., presence of missing curb cuts at a crossing, presence of a continuous sidewalk, and presence of a continuous sidewalk (both ends), using a camera with different zoom capabilities and lens options may also allow auditors to more reliably code BE features at the edge of the photo frame when using GigaPan®. The significantly higher IRR of identifying the presence of curb in the traffic and safety concept also suggests GigaPan® imagery has benefits over GSV. Thus, the enhanced detail capabilities of GigaPan®, coupled with the overall similarities in the reliability between GSV and GigaPan® across the various BE concepts, position GigaPan® as a potential, alternative tool to audit the BE.

### PA, Cohen’s kappa, and PABAK

In this study we used three measures of reliability: PA, Cohen’s kappa, and PABAK. The PA does not account for the probability of agreeing by chance and therefore Cohen’s kappa is the primary statistic used to rate reliability. However, Cohen’s kappa values may appear low when agreement is high because of very low or high prevalence or because of bias. In this study, PABAK was calculated to account for these factors. Across GigaPan® and GSV, PABAK values were consistently higher than Cohen’s kappa values for all but three BE features. However, PABAK should not be interpreted as measuring the same agreement as Cohen’s kappa—PABAK ignores the variation of prevalence across the BE features examined and assumes the absence of bias. In addition, PABAK has not been as thoroughly studied as Cohen’s kappa. Therefore, in congruence with previous researchers, we recommend using PABAK in addition to Cohen’s kappa to give a more complete picture of the data [30].

### Limitations

Both GSV and GigaPan® had imagery issues in our study. For example, our analytic sample was reduced because of GSV issues related to poor image quality, incomplete imagery, or non-existent street view imagery for the segment. Given the auditor is not able to capture or recapture GSV imagery, the BE could not be measured in these cases. These limitations are consistent with findings from a systematic review of GSV studies of neighborhood environments in North America, Europe, New Zealand, Australia, Japan, and Brazil [12]. Uniquely, the GSV imagery was updated for one segment during the audit process in our study. Furthermore, given the GSV imagery dates ranged from 2007 to 2016 and the GigaPan® imagery was from 2015, there is also temporal mismatch across methods for some segments. However, regardless of temporal alignment, the IRR results for GigaPan® and GSV are independent of each other because IRR is calculated within each method (GigaPan® or GSV). In other words, the IRR results of GigaPan® do not depend on the GSV imagery and vice versa.

Although GigaPan® has the potential to address some of these GSV imagery issues, it also has limitations. Issues unique to GigaPan® include: the image only captured a small fraction of the street segment, GigaPan® Stitch Software could not open some image files, incorrect images were taken by the field staff, and the images taken of both sides of the street did not correspond to each other. More segments were dropped from our sample due to GigaPan® issues than GSV issues, and the majority of the issues associated with GigaPan® were user error issues. This difference suggests there is greater potential for problems with obtaining GigaPan® imagery compared to GSV imagery.

When capturing the GigaPan® images or GSV imagery on segments where street parking existed, cars often blocked the point of view of the camera. This makes it challenging to see some features of the street and is consistently an issue in GSV studies internationally [12]. On the other hand, the user of GigaPan® maintains the ability to position the camera to strategically avoid or limit the interference of parked cars or other obstacles. Therefore, future research teams utilizing GigaPan® technologies should incorporate this solution into their GigaPan® procedures.

Another factor to consider when using GigaPan® is the quality of the camera. Although higher quality cameras increase supply costs for the project, higher quality cameras may improve the quality of the GigaPan® panoramas. The Canon® PowerShot S120 was used in this study, but future studies may consider using a camera with different zoom capabilities and lens options. Similarly, there are various GigaPan® apparatuses that exist. The GigaPan® Epic was used for this study. More costly GigaPan® apparatuses exist and, if used, may also improve the imagery details. Both cost and the necessity for detail must be considered when deciding what equipment would be most suitable for a similar study.

### Strengths

One of the biggest strengths of this reliability study was the large sample size – 106 unique street segments audited twice using GSV and GigaPan® imagery. In addition, data analysis for the study was rigorous and considered various reliability measures, as it included PA, Cohen’s kappa, and PABAK. Few studies incorporate all three data analysis methods. We also used a comprehensive audit tool which was tailored to the local context.

Another strength of this study is that both of the audit methods used (GigaPan® and GSV) were non-intrusive and did not place burden on community members. In fact, RAND partnered with local community organizations to train community partner data collectors who were from the Hill District or Homewood neighborhoods. This is particularly important given our study locations were low-income neighborhoods whose residents were predominately African American. The burden of health issues that trace back to the built and social environment in the USA are disproportionately experienced by communities of color and low-income communities [31]. Therefore, it is critically important to study how the microscale BE features in these environments relate to health and contribute to health disparities, but also strive to collaborate with the local community and limit additional burdens placed on the residents.

### *Implications and applications of GigaPan*®

GigaPan® has the ability to capture time-sensitive images. With GigaPan® images, the study team controls the image-capturing process in real time. This requires more planning than GSV, but resolves GSV issues related to missing imagery, varying imagery dates along audited segments, availability of less current imagery, and unexpected updates in street-level imagery after auditing processes have commenced. Furthermore, once the GigaPan® image is taken, the research team is responsible for the permanency/archival of the imagery. In GSV, historical imagery can be viewed but the research team cannot control imagery archival.

GigaPan® is also applicable to populations outside of academic researchers. Citizen science and community-based participatory research literature has demonstrated how individuals within low-income communities with little experience using technology can use innovative photography methodologies to gather information about the BE features in their neighborhoods [32, 33]. GigaPan® gives local residents the potential to study the BE in their own communities and work alongside a group or organization (e.g. researchers, community organizations, or local government agencies) to help collect data for their own purpose. Unlike GSV, the GigaPan® user is in direct control of when and where images are taken, allowing the user to take high-definition panoramic images of BE settings or features most relevant to the user’s plan/project. Therefore, GigaPan® panoramas may be beneficial to people involved in public planning processes, such as designing parks or public community areas.

GigaPan® may also be useful for studying streetscapes in communities where GSV imagery is outdated, limited, or nonexistent.It may be a particularly valuable tool for assessing the BE of streetscapes in: (1) countries with sparse GSV imagery availability, (2) rural communities; given rural areas tend to have less GSV coverage and the GSV imagery is updated less frequently than urban areas [6], and (3) studies in any setting across the urban–rural continuum that require present-day imagery.

Additionally, although not tested in this study, methods other than human auditors could possibly be used to assess features in GigaPan® imagery. Similar to how deep learning technologies have been used to extract information from GSV imagery, these same advancements in technology could potentially be utilized to extract information from GigaPan® imagery. Other GigaPan® researchers have also suggested that future studies should consider whether GigaPan® images can be analyzed using a computer algorithm or machine learning [34]. GigaPan® produces panoramas at the gigapixel scale (one billion pixels), which may further the capabilities of machine learning methodologies to accurately distinguish and classify features along the street. This development could extend the applicability of GigaPan® to fields of study that have been examined via a combination of GSV panoramas and machine learning. This could include using GigaPan® to study solar radiation [35, 36] or to estimate the demographic makeup of communities with potentially greater relevancy and frequency than the American Community Survey [37]. GigaPan® users could also be equipped with other assessment tools (e.g. air quality toolkits) to study the relationships between smaller spatial resolutions (e.g. street segments) and other environmental characteristics as has been done with GSV cars and air pollution monitors [38]. However, since GigaPan® is user-made photography, it does not inherently possess the large-scale applicability that GSV possesses. Future studies evaluating the streetscape using deep learning technologies should consider these points when deciding whether to use GigaPan® or GSV.

## Conclusions

Based on the results from this study, the audit tool selected (GigaPan® or GSV) for assessing BE features in future studies should be dependent on the specific goals of the research project. GigaPan® may be particularly well-suited for BE projects with study settings in areas where GSV imagery is nonexistent or updated infrequently. Additionally, the potential for enhanced, detailed imagery using GigaPan® will be most beneficial in studies in which BE details are a priority or smaller-scale BE features would be challenging to see in GSV imagery. Using GigaPan® may prove more costly than GSV, as the methodology requires people to travel to the destined location to capture imagery. In general, research should continue to explore the use of GigaPan® in evaluating the BE as the results from this study suggest GigaPan® is a reliable, alternative audit tool to GSV. Further reliability studies using machine learning to extract information from GigaPan® images could also be conducted.

In our study, the IRR of assessing BE features related to land use, traffic and safety, and public amenities was also impacted by the type of reliability statistic calculated. After accounting for the effects of bias and prevalence, reliability values were consistently higher across all BE features, except three. This was true of GSV audits and GigaPan® audits. This highlights the effect bias and prevalence has on our results, and the importance of using PABAK to supplement or expand upon Cohen’s kappa reliability findings. Future studies assessing the reliability of BE audit tools in measuring features with varying prevalence should consider using PABAK to supplement Cohen’s kappa. Such a statistical approach provides additional perspectives on the reliability of the BE audit tools being evaluated.

## Data Availability

The datasets used and/or analysed during the current study are available from the corresponding author on reasonable request.

## Abbreviations

BE: Built environment
BTG-COMP: Bridging the Gap Community Measures Project
CI: Confidence interval
DO: Direct observation
GP: GigaPan®
GSV: Google Street View
IRR: Inter-rater reliability
PA: Percent agreement
PABAK: Prevalence-adjusted bias-adjusted kappa
PR: Average prevalence
USA: United States
